# Visual Perception in Expert Athletes: The Case of Rock Climbers

**DOI:** 10.3389/fpsyg.2022.903518

**Published:** 2022-07-14

**Authors:** Noel Marcen-Cinca, Xavier Sanchez, Sofia Otin, Cristina Cimarras-Otal, Ana Vanessa Bataller-Cervero

**Affiliations:** ^1^Department of Health Sciences, Universidad San Jorge, Villanueva de Gállego, Spain; ^2^Université d’Orléans, Complexité, Innovation et Activités Motrices et Sportives (CIAMS), Orléans, France; ^3^Université Paris-Saclay, Complexité, Innovation et Activités Motrices et Sportives (CIAMS), Orsay, France; ^4^School of Health and Welfare, Halmstad University, Halmstad, Sweden; ^5^Department of Ophthalmology, Miguel Servet University Hospital, Zaragoza, Spain; ^6^Aragon Health Sciences Institute (IIS Aragón), Zaragoza, Spain; ^7^Department of Applied Physics, University of Zaragoza, Zaragoza, Spain

**Keywords:** contrast sensitivity, expertise, visual acuity, visual field, visuo-motor development

## Abstract

The purpose of the present study was to examine the visual perception system in expert climbers through a psychophysical optical test in a cross-sectional study. Twenty-seven male participants with an International Rock Climbing Research Association (IRCRA) best on-sight lead skill level ranging between 18 and 27 and a best red-point level ranging between 18 and 29 completed a series of psychophysical optic tests assessing their visual field, visual acuity, and contrast sensitivity. Climbers were divided by their best red-pointed lead level, and, following IRCRA recommendations, two groups were created: an advanced group (IRCRA redpoint level between 18 and 23), and an elite-high elite group (IRCRA redpoint level between 24 and 29). The elite group presented more training days per week (5.25 ± 1.28), best on-sighted lead level (24.63 ± 1.92 IRCRA), and best red-pointed lead level (26.63 ± 2.56 IRCRA) than the advanced group (3.67 ± 0.91 training days per week, 19.50 ± 1.04 IRCRA on-sighted level and 20.67 ± 1.57 IRCRA red-pointed level). Better visual perception outputs were produced by the group of elite climbers in visual field tests; no differences were observed between the two groups for visual acuity and contrast sensitivity tests. Overall, findings indicate that best climbers performed better at the visual perception tasks that tested their visual field. Such better perception from best climbers is discussed given (1) the greater time they spend coercing the visual system during practicing climbing and (2) the specific complexity of the stimuli as they are confronted to harder routes where holds are less perceptible and the time to find best hold sequences is constrained.

## Introduction

The sport of climbing, included in Tokyo 2021 Olympic Games and in the Olympic program for the forthcoming Games, has been examined from physiological (España-Romero et al., 2012), anthropometric (Laffaye et al., 2015), biomechanical (Guo et al., 2019), psychological (Jones and Sanchez, 2017) and nutritional perspectives (Potter et al., 2019). A critical aspect outlined by most when it comes to optimizing climbing performance is that of route previewing (Pezzulo et al., 2010; Sanchez et al., 2019). Such a pre-ascent climbing route visual inspection is defined as the ability to visualize and remember climbing hold configurations and to interpret the movement sequences (Sanchez et al., 2012). To better understand such a key process, the present study assessed visual perception parameters in expert climbers.

When it comes to the perceptual constraints, two different climbing styles can be distinguished given previous knowledge of the route; on-sight and red-point climbing (Draper et al., 2011). An on-sight ascent is performed when a route is completed on the first attempt without any prior knowledge of its features. The hardest routes are typically climbed in red-point style, in which the climber successfully completes the route after two or more attempts, so the climber already knows the best hold scheme. The lack of knowledge of hold features and sequence has been identified as a key impediment to optimal performance when attempting an on-sight ascent (Ferrand et al., 2006; Sanchez et al., 2012).

Recent studies have associated route information gathering—hold features and movement combinations—with exploratory actions. Indeed, the ability to find best options relative to constraints and capabilities influences climbers ascent performances (Sanchez et al., 2012; Orth et al., 2017b). Research has also examined climbers’ exploratory actions (Button et al., 2018), with findings linking route exploration to climbing performance improvements (Seifert et al., 2015). Previewing both the physical characteristics of the route as well as the way holds are best to-be-grasped and used has been shown to be a factor for success; it allows climbers to determine the time needed to hold a difficult position, climb upwards by enchaining different holds, and ultimately affects the speed of the climb overall (Sanchez et al., 2012; Seifert et al., 2015). In that line, climbers have expressed that failing to identify the correct strategy and hold order may result in a fall during the ascent (Boschker et al., 2002). The viability to grasp a hold may differ depending on its rugosity, adherence and depth (Amca et al., 2012). Knowledge generated from climbing experience may help to complete this information needed. Thus, an experienced climber may visually perceive the hold’s rugosity and texture before reaching it. Whitaker et al. (2020) have recently suggested that skilled climbers may have a better tuned perceptual system. The visual system would indeed be the first to intervene in such an information gathering process.

Vision is the capacity to both recognize and interpret the environment, which involves several physical and biochemical processes. The study of vision includes visual system (hardware) and visual strategy (software). Hardware is related to the optometric features of the visual system whereas software is related to the analysis and coding of the visual data (Williams et al., 1999). Whilst the latter has been examined within the sport of climbing (Nieuwenhuys et al., 2008; Sanchez et al., 2012), the study of the former is scarce. Previous research, in general, suggests examining contrast sensitivity in combination with visual acuity and visual fields to gather a comprehensive picture on how well one’s visual system functions (Elliot and Flanagan, 2007; Hadlow et al., 2018). Such testing provides useful information about real-world vision and control of body balance. Visual field refers to the total area in which it is possible to detect and react to stimuli in the peripheral vision as the eyes focus on a central point. The photoreceptors are distributed over the retina, and the purpose of visual field assessment is to rate the thresholds of light sensitivity of these receptors, measured in decibels (dB). The threshold indicated the minimum light intensity that the receptors were able to capture. In the study, the visual fields were divided into 54 points that were grouped into two major areas: The upper visual field, and the lower visual field. Visual acuity represents a complex function that can be defined as the combination of three capacities: (1) The smallest spatial unit that the visual system is able to discern, (2) the minimum distance between two objects that can be distinguished as separate, and (3) the ability to recognize the details of an object (Bailey and Lovie-Kitchin, 2013). Visual acuity provides information about the limits of an individual’s vision, but it does not provide information about what happens within these limits. Contrast sensitivity represents the capacity of the visual system to filter and process figures and background information under varying conditions (Elliot and Flanagan, 2007).

Climber’s movements are thought to be highly dynamic (Wright et al., 2018). As climbers ascend, their perspective changes, as does their perception of the holds they use to climb up the route; that is, the visual system is persistently perceiving and processing information (Boschker et al., 2002; Sanchez et al., 2012; Seifert et al., 2015). Vision provides predictive information for prospective control of movement pattern (Patla, 1991). As previous findings suggest, the vision allows to scan the environment and ascertain the relevant information (Williams et al., 2004; Broadbent et al., 2015).

Given the functionality of the visual perception system in sport climbing, the present study aimed at gaining knowledge and understanding in this area by assessing, through a series of psychophysical optic tests, expert climbers’ visual perception hardware system. Given the lack of research in this area, we adopted an exploratory, cross-sectional design whereby it was suggested that elite climbers would perform better at the psychophysical optic tests than advanced climbers. That is, the visual hardware system would be further developed in elite climbers.

## Materials and Methods

The protocol of the present study was approved by the Ethics Research Committee of the first author’s regional government (C.I. PI3/0100). Every procedure was conducted in accordance with the principles of the Declaration of Helsinki. Each participant was informed of the nature of the study, the voluntariness of the participation and of any potential adverse effects and signed an informed consent form.

### Participants

Twenty-seven climbers with a self-reported outdoor sport climbing redpoint lead level (Draper et al., 2011) ranging between 7a + and 9a on the French Rating Scale of Difficulty (F-RSD) participated. We used F-RSD as it is the scale our participants were used to report climbing ability levels. However, to statistically process the data and following recommendations from the climbing research community, the F-RSD grades were transformed into climbing levels of the International Rock Climbing Research Association (IRCRA; Draper et al., 2016). Thus, our study sample’s redpoint IRCRA levels ranged between 18 and 29.

In this cross-sectional study, visual parameters were examined and participants were grouped as a function of their best redpoint ascent. The inclusion criteria were: (a) To be over 18 years old with an outdoor climbing best redpoint lead within the month prior to testing of at least 18 IRCRA; and (b) to possess a healthy, free of anomalies anatomo-structural integrity of the retina (retinal nerve fiber layer was evaluated by Optical Coherence Tomography; Spectralis^®^, Heidelberg Engineering Inc. Carlsbad, CA, United States). See sample demographic characteristics and climbers’ years of experience as well as IRCRA levels in Table 1.

**TABLE 1:** Mean (SD), *p*-values and Effect Size (ES) for the demographic, anthropometric and ophthalmologic data.

	Advanced mean (*SD*)	Elite-high mean (SD)	*P*-value	ES
Age (years)	32.22 (6.93)	27.27 (5.95)	0.15	0.2
Age start climbing (years)	17.72 (4.76)	14.88 (6.03)	0.37	0.12
Years training (years)	7.78 (5.42)	10 (4.72)	0.14	0.2
Days/week climbing	3.67 (0.91)	5.25 (1.28)	0.01*	0.38
Best on-sight lead (IRCRA scale)	19.50 (1.04)	24.63 (1.92)	0.00*	0.55
Best red point lead (IRCRA scale)	20.67 (1.57)	26.63 (2.56)	0.00*	0.55
VA100	–0.15 (0.08)	–0.20 (0.05)	0.26	0.15
VA2.5	0.24 (0.11)	0.18 (0.1)	0.22	0.17
VA1.25	0.37 (0.09)	0.29 (0.13)	0.15	0.2
CS3	1.81 (0.13)	1.86 (0.14)	0.38	0.12
CS6	2.07 (0.15)	2.14 (0.08)	0.18	0.18
CS12	1.82 (0.12)	1.8 (0.07)	0.57	0.08
CS18	1.4 (0.15)	1.4 (0.16)	0.91	0.02

*Advanced, IRCRA advanced ability group; Elite-High, IRCRA Elite and High Elite ability; VA100, Visual Acuity 100% contrast; VA2.5, Visual Acuity 2.5% contrast; VA1.25, Visual Acuity 1.25% contrast; CS3, Contrast sensitivity 3 cycles/degree; CS6, Contrast sensitivity 6 cycles/degree; CS12, Contrast sensitivity 12 cycles/degree; CS18, Contrast sensitivity 18 cycles/degree.*

*Shows significant differences between Advanced and Elite-High groups. P-values of 0.00 represents P-values < 0.005.

### Measurements and Procedures

Participants completed a series of psychophysical optics tests at the Visual Function Unit of a university hospital. The exploratory protocol was carried out by an academic expert in optic and ophthalmologic research.

#### Visual Field

Two visual field (VF) tests were carried out with our sample of climbers: that is, the contrast sensitivity to coarse vertical grating targets and the white-on-white 30–2 automated perimetry. The contrast sensitivity to coarse vertical grating targets was performed using the frequency-doubling technology perimeter (FDT; Matrix Frequency-Doubling Perimeter, Carl Zeiss Meditec, Dublin, CA, United States). The FDT perimeter displays sine waves that vary stimuli in temporal frequencies at 25 Hz and spatial frequencies at 0.25 cycles/deg. The first stimulus is characterized by a low temporal frequency that increases progressively until it becomes not visible; that is, it reaches the temporal threshold for a given spatial frequency and contrast. Then, the spatial frequency is increased until another threshold is reached. The process continues until a threshold for the combinations of spatial and temporal frequencies for each specific photoreceptor group visual system is established (Anderson and Johnson, 2003). The white-on-white 30–2 automated perimetry (SITA-Standard strategy) was performed using the Heidelberg Edge Perimeter (HEP; Heidelberg Engineering, Germany). HEP assesses the threshold of light sensitivity of a white point on a white background for all photoreceptor groups (Kaczorowski et al., 2015).

#### Visual Acuity

Three outcome measures were gathered utilizing the Bailey-Lovie charts, and scored in logMAR units (logarithm of the Minimum Angle of Resolution): high contrast 100% (VA100), low contrast 2.5% (VA2.5), and low contrast 1.25% (VA1.25). These charts have standardized spacing arrangements between optotypes (letters)—charts have the same number of optotypes in each row, a constant ratio of size progression, and the spacing between optotypes within rows and between rows is proportional to the given optotype size (see full details in Bailey and Lovie-Kitchin, 2013).

#### Contrast Sensitivity

Contrast sensitivity was evaluated with the CSV 1000E test (Vector Vision, Dayton, OH, United States), which assesses the whole contrast sensitivity function from the lowest to the highest spatial frequencies. The instrument presented a series of photocells that automatically monitored and calibrated the instrument light level. The test was composed of eight contrast levels. Across the first four levels, the contrast decreased by steps of 0.17 logarithm units, while it decreased by steps of 0.15 logarithm units across the last four levels. The test was performed at 2 m distance.

Four outcome measures were gathered in relation to four spatial frequencies in the translucent chart: 3 (CS3), 6 (CS6), 12 (CS12), and 18 (CS18) cycles/degree. Each spatial frequency was presented on a separate row of the test. Each row contained 17 circular patches that were 3.8 cm in diameter. The first patch in the row presented a very high contrast grating. The remaining 16 patches appeared in eight columns. In each column, one patch presented a grating, and the other patch was blank. The patches that presented gratings decreased in contrast from left to right across the row. Participants were asked to observe the first patch and then told to look for the grating pattern in each column. While reading across the row, they had to indicate whether the grating appeared at either the top patch or the bottom patch of each column. The contrast threshold was taken as the last correct answer. This test determined the contrast function curve and the behavior of the visual system. Contrast sensitivity provides information about real-world vision, including balance control or probability of falling.

### Statistical Analysis

Following recommendations from the field that advise to use climbers’ skill level as an indicator of expertise instead of years of experience (see Whitaker et al., 2020), participants in the present study were grouped based on their redpoint climbing skill level. The grouping was based on the IRCRA ability grouping. Following IRCRA recommendations, climbers were grouped in either as advanced (IRCRA 18–23) or elite—high elite (24–29) climbers.

Descriptive statistics [mean ± standard deviation (SD)] were calculated. Normal distribution of continuous variables was tested using the Shapiro-Wilk test, Kolmogorov-Smirnov test. All statistical procedures were completed on IBM™ SPSS™ Statistics (version 21, IBM Corporation, Somers, NY). A first-level analysis that compared visual acuity, contrast sensitivity, and visual fields between groups was carried out using independent *t*-test when data was normally distributed and U Mann-Whitney when data was not normally distributed. The magnitude of each change was assessed using Cohen *d* effect size (Cohen, 1988) (ES; *d* ≤ 0.2, small; 0.5–0.79, moderate; ≥ 0.8, strong). Significance level was set at *p* ≤ 0.05.

## Results

Differences between groups were observed for both climbing days per week, best on-sighted lead level and best red-pointed lead level. No differences were found for chronological age, age participants began to practice and years of training (see Table 1).

**FIGURE 1:**
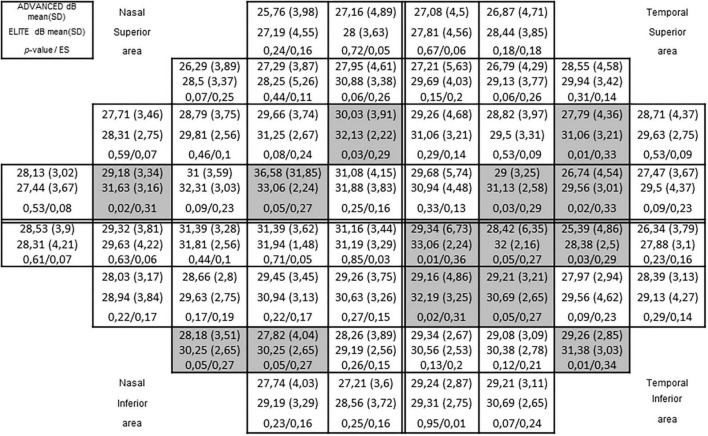
Frequency-doubling technology perimeter (FDT) visual field results between advanced and elite groups. The complete sector shaded cells represent significant differences (*p* < 0.05). *P-*values of 0.00 represents *P-*values < 0.005.

**FIGURE 2:**
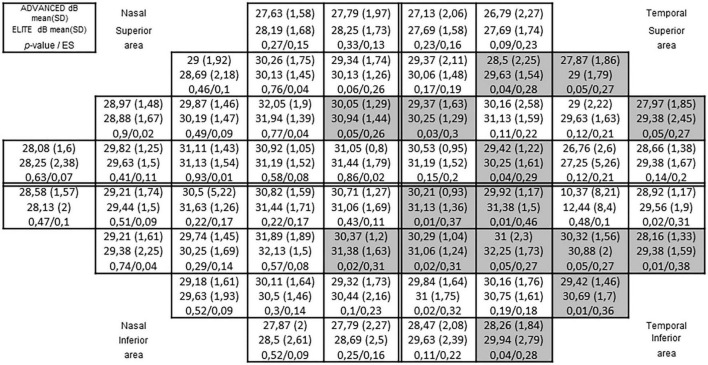
Heidelberg edge perimeter (HEP) Visual field results between advanced and elite groups. The complete sector shaded cells represent significant differences (*p* < 0.05).

With regards to the visual perception parameters assessed, differences were found between groups in the upper visual field and the lower visual field for the test FDT (see Figure 1), and for the test HEP (see Figure 2). Both figures provide an overview of the complete 54 visual field points, which are divided into two upper areas comprising 27 points and two lower areas comprising 27 points. 14 FDT points presented significant differences, ranging between ES = 0.27 and ES = 0.36 and 15 HEP points presented significant differences, ranging between ES = 0.27 and ES = 0.46. In all cases, but one FDT point, better visual perception scores were observed for the elite—high elite group. With regards to the remaining visual perception parameters, no differences between groups were found for visual acuity or contrast sensitivity (see Table 1).

## Discussion

The present study examined visual perception parameters in expert climbers. Findings showed that better climbers—the elite group—performed better at the tasks testing their visual field. These results may suggest that best climbers possess better perception of visual field. The group of elite climbers comprised those with more training days per week, and who had climbed the hardest routes. Thereby, we can assert that these climbers have been exposed, in general, to a higher number and variety of climbing stimuli than the advanced group.

The visual information about the extra-personal space has been related to route finding and hold identification. It has been observed that climbing style affects the number and duration of sight fixations in the upper visual field. For example, climbers look more frequently and for longer times overhead, searching for the next hold to reach (Sanchez and Dauby, 2009; Seifert et al., 2015). In the present study, climbers who have been exposed to a higher number and variety of climbing stimuli, and who have climbed harder routes showed some better perception in the visual field. This better perception could be mediated by the neural process underlying the ventral pathway. As climbers face more climbing stimuli (i.e., more and harder routes) it may facilitate the neural process in this neural stream (Sheth and Young, 2016). Nevertheless, due to the cross-sectional nature of the study, one cannot establish whether such better perception system (visual hardware) is due to such environmental demands and challenges, even though past research associated the development of the visual hardware with the environment in which one develops and interacts (Schoups et al., 2001).

With regards to visual acuity, no differences between the two groups were found; however, climbers in our study showed a similar trend to that shown by other athletes (Zimmerman et al., 2011). Indeed, Zimmerman et al. (2011) found that similar age expert baseball players showed –0.16 logMAR (advanced climbers scored –0.15 logMAR; elite climbers scored –20 logMAR). They also suggested that these baseball players’ visual acuity may be superior to those in the general population. As far as the authors know, there is no established normative data for visual field tests in healthy and young population. Ve Ramesh et al. (2007) have described normative data comparing patients with a control group of healthy subjects. The control group in their study showed a mean threshold sensitivity in the frequency-doubling technology perimeter test that ranged between 26 dB in peripheral visual field, and 29 dB in central visual field. Climbers’ thresholds, especially in the elite group seem higher, ranging between 28 and 33 dB. Ve Ramesh et al. (2007) applied a variant of the frequency-doubling technology perimeter test, and their control group was around 20 years older than the climbers of our study. Though, it is worth mentioning that a decrease between 0.6 and 0.9 dB per decade of age is suggested (Adams et al., 1999).

Previous studies have shown that expert climbers have better visual strategies than lower-level climbers (Boschker et al., 2002; Sanchez et al., 2012; Seifert et al., 2014a). Whitaker et al. (2020) suggested that skilled climbers are “more sensitive to the properties of their environment that specify affordances” (Whitaker et al., 2020, p. 507). Affordances have been defined as opportunities for action that a subject is able to perceive (Gibson, 1979). As mentioned earlier, it has been suggested that expert climbers better perceive the wall functionality, compared to novices (Boschker et al., 2002). Whitaker et al. (2020) discussed the advantage in perception found in expert climbers in terms of a more tuned perceptual system (Whitaker et al., 2020). When climbing, better perception of the shape, depth, contrast and other route details are considered as assets that may help climbers to perform a difficult movement, a *crux*, and climb more dynamically, with fewer and shorter stops for exploratory movements (Sanchez et al., 2012). Such stops may lead to an increase in the overall isometric work and thereby increase energy expenditure, and ultimately deteriorate climbing performance (España-Romero et al., 2012) and climbing fluency (Nieuwenhuys et al., 2008; Sanchez et al., 2012; Seifert et al., 2015; Orth et al., 2017b). Isometric work can also alter the forearm blood flow, another aspect that could decrease climbing performance (Fryer et al., 2016).

Perceptual and cognitive processing has been barely studied (e.g., Seifert et al., 2014b; Whitaker et al., 2020). Seifert et al. (2014b) suggested that perceiving the features of holds could improve perceiving affordances. According to Orth et al. (2017b), more successful climbers are most effective in how they explore new routes. Seifert et al. (2017) proposed expert climbers display better perceptual “attunement” that enables them to better perceive the environmental information needed to use the climbing holds with accuracy. Before applying any strength to the hold, an expert climber may adjust according to how the hold is perceived. Such behavior may enhance the way holds are grasped (Orth et al., 2017a; Fuss et al., 2020). This mechanism has been studied in ice climbing; expert ice climbers showed a better perceptual performance for acoustic, haptic and visual information when compared to non-experts (Seifert et al., 2014a). Interestingly, Orth et al. (2017a) suggested that an important difference between diverse skill level climbers was the perceptual information that they pay attention to; that is, the affordances available.

Visual strategies such as route finding have been studied and associated with sport climbing performance (Boschker et al., 2002; Sanchez et al., 2019). Given the findings of the present study, we suggest that the visual system may, at least partially, influence somehow the visual strategies climbers will employ. It has been recently suggested that differences in visual search between experts and no-experts may be explained by experts’ superior use of their peripheral vision (Mitchell et al., 2020). Climbers would benefit from a training program based on peripherical vision to be able to obtain more and/or better information. Probably, the best stimuli for this training routines would be focus on contrast to low sensitivity to be able to better appreciate the holds features.

## Strengths and Limitations

To the best of the authors’ knowledge, this study is the first of its kind to examine the visual hardware in climbing. The study of visual perception in new disciplines such as sport climbing is warranted given its specific in-nature type of activity. The sport of climbing requires route examination, which is critical for the discipline for both performance and safety issues.

Whilst the present study is a first step toward a better understanding of the perception system in climbing, some limitations shall be addressed. Firstly, the cross-sectional nature of the design entail a cautious interpretation of findings; experimental studies are warranted to test whether given visual training programs may ultimately influence climbing performance, as it has been shown for other sports (Seya and Mori, 2007). Secondly, optic tests conducted in the present study were not climbing-specific. It would be interesting to examine visual perception further using more climbing-specific stimuli, and including testing whilst actual performance takes place (e.g., on a climbing treadmill). Lastly, we must acknowledge the increase in familywise error rate across the statistical analysis, which was not controlled for.

Overall, we consider the present research relatively preliminary and encourage further replication. To better understand peripheral perception in climbers it is necessary to (a) perform similar analysis while measuring dynamic visual acuity, as it has been proven to obtain better results than static visual acuity (Souissi et al., 2021); and (b) evaluate the visual fields stimuli more similar to climbing holds, where the perception of depth and contrast of different hold-types could be analyzed (e.g., by using a climbing treadmill, or an eye-movement registration system (Button et al., 2018; Mitchell et al., 2020). The ability to navigate a climbing route is a high cognitive function and as such, is necessary to link the perception performance to motor action (Wolbers and Hegarty, 2010; Whitaker et al., 2020) and even in stress situations, specific of this sport (Broadbent et al., 2015).

## Conclusion

The present study was the first to investigate visual perception amongst expert rock climbers through a series of psychophysical optic tests. Interestingly, climbers with more experience and higher on-sight and red-point best lead skill levels better perceived the stimuli in the visual field. Such better perception may be explained by (1) the greater time spent coercing the visual system during the process of climbing and (2) the complexity of the stimuli climbers encounter when climbing harder routes, in which holds are less perceptible and time to find the best hold sequence is constrained. Further studies on visual fields in climbing may contribute to a better understanding on how expert climbers perceive hold characteristics, thus influencing positively route previewing and, ultimately, actual climbing performance.

## Data Availability Statement

The raw data supporting the conclusions of this article will be made available by the authors, without undue reservation.

## Ethics Statement

The studies involving human participants were reviewed and approved by the Ethics Research Committee of Aragón (CEICA) (C.I. PI3/0100). The patients/participants provided their written informed consent to participate in this study.

## Author Contributions

NM-C and SO contributed to the conception of the study and performed the experiment. NM-C, XS, AB-C, and CC-O performed data analysis and contributed to the production of a first draft of the manuscript. NM-C and XS wrote subsequent drafts and produced the final version of the manuscript. All authors of the present study contributed to the work submitted and approved the final version for publication.

## Conflict of Interest

The authors declare that the research was conducted in the absence of any commercial or financial relationships that could be construed as a potential conflict of interest.

## Publisher’s Note

All claims expressed in this article are solely those of the authors and do not necessarily represent those of their affiliated organizations, or those of the publisher, the editors and the reviewers. Any product that may be evaluated in this article, or claim that may be made by its manufacturer, is not guaranteed or endorsed by the publisher.
